# Mitochondrial Dysfunction and Therapeutic Targets in Auditory Neuropathy

**DOI:** 10.1155/2020/8843485

**Published:** 2020-08-28

**Authors:** Baoyi Feng, Chenxi Jin, Zhenzhe Cheng, Xingle Zhao, Zhuoer Sun, Xiaofei Zheng, Xiang Li, Tingting Dong, Yong Tao, Hao Wu

**Affiliations:** ^1^Department of Otolaryngology-Head and Neck Surgery, Shanghai Ninth People's Hospital, Shanghai Jiaotong University School of Medicine, No. 639, Zhizaoju Road, Shanghai 200011, China; ^2^Ear Institute, Shanghai Jiaotong University School of Medicine, No. 115, Jinzun Road, Shanghai 200011, China; ^3^Shanghai Key Laboratory of Translation Medicine on Ear and Nose Disease, No. 115, Jinzun Road, Shanghai 200011, China; ^4^Biobank of Ninth People's Hospital, Shanghai Jiao Tong University School of Medicine, No. 115, Jinzun Road, Shanghai 200011, China

## Abstract

Sensorineural hearing loss (SNHL) becomes an inevitable worldwide public health issue, and deafness treatment is urgently imperative; yet their current curative therapy is limited. Auditory neuropathies (AN) were proved to play a substantial role in SNHL recently, and spiral ganglion neuron (SGN) dysfunction is a dominant pathogenesis of AN. Auditory pathway is a high energy consumption system, and SGNs required sufficient mitochondria. Mitochondria are known treatment target of SNHL, but mitochondrion mechanism and pathology in SGNs are not valued. Mitochondrial dysfunction and pharmacological therapy were studied in neurodegeneration, providing new insights in mitochondrion-targeted treatment of AN. In this review, we summarized mitochondrial biological functions related to SGNs and discussed interaction between mitochondrial dysfunction and AN, as well as existing mitochondrion treatment for SNHL. Pharmaceutical exploration to protect mitochondrion dysfunction is a feasible and effective therapeutics for AN.

## 1. Introduction

Hearing loss is one of the most crucial public health issues. According to the 70th World Health Assembly (WHA), 360 million people are suffering from auditory dysfunction in the world, accounting for 5% of the world's population. Besides, more than 1000 million juveniles are risky to hearing disorder [1]. Auditory dysfunction causes speech communication barrier, cognitive disorder, psychological isolation, and inferiority but also brings a heavy burden on family and society. SNHL is the major type of deafness, representing damage in the inner ear or auditory nerves that travel from the ear to the brain [2]. The etiology of deafness is complex, and SGNs draw more and more attention recently [3].

AN or auditory disease was first proposed by Kaga et al. [4] and Starr et al. [5] in 1996, referring to an acquired disorder characteristic of slight hearing impairment with wave I-III absence of auditory brainstem response (ABR) and speech recognition disorder, while distortion product otoacoustic emission (DPOAEs) and cochlear microphonic potential (CMs) did not change. AN may present as a sole clinical phenotype or just be one of the symptoms in systematic diseases like hereditary sensorimotor neuropathies (HSMN) or other demyelinating diseases. Pathology evidence demonstrated auditory nerve damage and loss of inner hair cells (IHCs) and ribbon synapses in AN. AN could be aroused by hereditary defects; for instance, mutation of genes encoding otoferlin or vesicular glutamate transporter 3 was found to induce IHC presynaptic and postsynaptic dysfunctions, respectively. And exogenous damage is another key contributor to be reckoned with, including noise exposure, ototoxic drugs, hyperbilirubinemia or thiamin deficiency in infant, or presbycusis [6].

Mitochondrion dysfunction is a major reason for neuropathy. Mitochondria, serving as the engines of eukaryotic cells, participate in cellular energy metabolism, ROS generation, calcium homeostasis, and apoptosis. Mitochondria exhibit special dynamic nature, with feature of pluralistic morphology and great interconnectivity, which determine their function and network structure. Mitochondrion dysfunction is a key reason in aging and neurodegeneration like Alzheimer's disease (AD), amyotrophic lateral sclerosis (ALS), Charcot-Marie-Tooth disease (CMT), and optic atrophy [7]. Additionally, association between mitochondrial biology and optic neuropathies were also detailedly illustrated by pathology and relevant molecular and therapeutic targets. Patients with neuropathy including myoclonic epilepsy with ragged-red fibers (MERRF); mitochondrial encephalomyopathy, lactic acidosis, and stroke-like episodes (MELAS); Charcot-Marie-Tooth disease type 2A (CMT2A); and HSMN caused by mitochondrial dysfunction [8] were also observed suffering from sensorineural hearing loss [9, 10]. The mutation of optic atrophy 1 (OPA1), a key protein related to mitochondrial fusion, was proved to cause syndromic autosomal dominant optic atrophy (DOA+) with auditory dysfunction [11], which reveals to the potential association between auditory nerves and mitochondria in the development of hearing disorders.

Thus, it is of great significance to explore mitochondrial mechanism of auditory neuropathy and may identify the therapeutic target of auditory neuropathy. In this review, we supply a brief introduction in the mitochondrial structure and function which is correlative to auditory neuropathy and illustrate the potential mechanism between mitochondrial dysfunction and auditory neuropathy. Ultimately, we enumerate the effective therapies targeting mitochondrion dysfunction in AN.

## 2. Mitochondrial Genome and Function

### 2.1. Mitochondrial Genome

Mitochondrial DNA (mtDNA), which is a mitochondrion-specific genetic system, exists as double-stranded circular molecule with a length of 16569 bp in human. Composed of a heavy strand and a light strand, mtDNA encodes 2 rRNAs, 22 tRNAs, and 13 subunits of the proteins and complexes in respiratory chain including COX I, II, and III and ATP synthase [12], illustrating its crucial role in oxidative phosphorylation (OXPHOS). Plenty of mutations in mtDNA are associated with anomalous OXPHOX. The diversity of mtDNA mutation was observed in neurodegeneration due to the neurons vulnerable to energy supply, especially during aging [13]. The deletion of mtDNA aggravated age-related hearing loss at 12 months of Fischer 344 male rats [14], while D257A and T7511C mutation in mtDNA accelerated the progression of age-related hearing loss and degeneration of HCs and SGNs [15, 16]. Moreover, mitochondria are sensitive to ROS since excessive ROS impedes unfolding of protein; therefore, ROS induce mtDNA mutation [17].

MtDNA is of maternal inheritance, and the copy number of mtDNA reaches nearly 1000 in majority of cells, hundreds of times as nuclear DNA genomes. Additionally, mitochondrial biogenesis or heteroplasmy occurs independently in cell division, allowing mutated mtDNA distributed unevenly in subcultured cells without efficient repairment, which was observed in most of the mitochondrial disease [18].

### 2.2. Mitochondrial Homeostasis

Mitochondrion is an organelle with high interconnection and constant movement, forming cellular networks through a dynamic process. Mitochondrial homeostasis refers to the steady status of the mitochondrial network structure between mitochondrial biogenesis and degradation, including mitochondrial fusion and fission, mitophagy, and trafficking. Disorders of mitochondrial homeostasis have been found in aging and plenty of age-related diseases like neurodegeneration and cardiovascular disease.

Mitochondrial biogenesis is a renewed process of mitochondria by growth and division, associated with protein synthesis, import, and assembly under the guidance of nuclear DNA and mtDNA [19]. Fusion acts on mitochondrial remodelling, modulated by proteolytic processing and PINK1-dependent ubiquitination. Fission allows the extraction of damage segment and quality control of mitochondria, which depends on several critical proteins owning highly conserved dynamic GTPase domain. Mitofusins 1 and 2 (Mfn1 and Mfn2) are located in the outer mitochondrial membrane, and Opa1 was anchored in the inner mitochondrial membrane. Fusion and fission are also involved in the process of mitophagy with the help of dynamic-related protein 1 (Drp1), a crucial mediator of mitochondrial fission assembled with Fis1 after posttranslational modifications, which could accelerate mitochondrial division [10, 20]. Apoptosis could be activated by means of regulating proapoptotic factors delivered and expressed in the cytoplasm, such as cyto-c and Bcl-2 [21–24].

Mitophagy is a vital process for mitochondrial quality control that could eliminate impaired mitochondria in time. When mitochondrial membrane potential vanished, PINK1 aggregated on the mitochondrial outer membrane with phosphorylation of Mfn2 and Parkin, inducing ubiquitination of multiple downstream proteins. Finally, impaired mitochondria were separated [10]. Besides, mitochondrial renewal and long-distance energy supply rely on mitochondrial trafficking orthodromic and antidromic. It is essential to neuron that their survival leans more heavily on mitochondrial trafficking than other cells for its high energy consumption and unique cellular morphology. Studies demonstrated fundamental significance to mitochondrial trafficking of motor/adaptor complex composed of kinesin, dynein, Milton, and Miro [25]. Mitochondrial trafficking mechanism in neurodegeneration has been widely studied in AD, Parkinson's disease, Huntington's disease, and amyotrophic lateral sclerosis (ALS) [26].

### 2.3. Mitochondrial Energetic Metabolism

As a cellular energy organ in eukaryote, mitochondria play vital roles in energy metabolism and ATP production through two essential process, the citric acid cycle (TCA) and OXPHOS. The TCA cycle is a critical task in aerobic respiration of eukaryotes as well as an ultimate metabolic step of carbohydrates, fats, and proteins. The close loop initiates with citrate production as acetyl-coenzyme A drifted into TCA cycle and ends as fumarate converted into oxaloacetate, in which electron carriers NADH and FADH2 were manufactured and further participating in electron transfer to electron transport chain (ETC) [27]. The OXPHOS system operates as the launch of ETC. ETC is situated in the inner membrane of mitochondria (IMM), performing functions in conveying electrons through complex I-III, cyto-c, and complex IV successively to convert oxygen to water and driving proton gradient production. Coenzyme Q (CoQ) is the key intermediate electron transporter of this process. With the actuation of proton gradient, ATP is released via ADP phosphorylation through complex V (ATP synthase). Nonetheless, there is still a bit of energy that remained besides the portion consumed by ATP synthesis, as the protons are able to leak across IMM and induce ROS generation to mitochondrial matrix via complexes I and III to a great extent [28]. ROS is an indispensable regulator for normal cellular activities covering intercellular communication as the secondary messenger, proliferation, differentiation, and apoptosis, while excessive accumulation of ROS might lead to oxidative damage, cell death, and diseases like cancer as well as neurodegeneration [29].

Besides, mitochondria also impact apoptosis and regulate calcium flux through mitochondrion-associated ER membranes, which not only act as the second messenger but also are essential to neurotransmitter release like glutamine [30]. As there is a high consumption of energy, normal activities of neuron are bound up with functional mitochondria, including auditory nerves.

## 3. Mitochondrial Dysfunction in Auditory Neuropathy

### 3.1. Auditory Neuropathy and the Role of SGNs in Auditory Pathway

Neuropathy is a common pathology in SNHL, related to age-related hearing loss and noise-induced hearing loss. Significant SGN degeneration followed by age is observed in apical and basal turns of both human and other mammals' cochlea, while inner or outer hair cells (OHCs) remain existing [31–34]. In Alzheimer's disease (AD), a study found significant loss of SGNs, rather than HC death, which could be found in the cochlea of both 9- and 12-month-old 3xTg-AD model mice [35]. Meanwhile, it was demonstrated that swollen cochlear nerve dendrites were seen in the first 24 h after noise exposure which could lead to temporary threshold shifts (TTS), without HC loss [36]. DPOAE threshold shifts were mild, suggesting that neuropathy and loss of ribbon synapse also contributed to the hearing loss prior to OHC damage. OHCs recovered 2 weeks after exposure, but delayed neurodegeneration was still observed for a long time [37]. In addition to aggravation of ABR threshold and aberrant compound potential of spiral ganglion, impaired SGNs also conduced to degraded precision of acoustic signal encoding and abnormal speech recognition [6].

**Figure 1:**
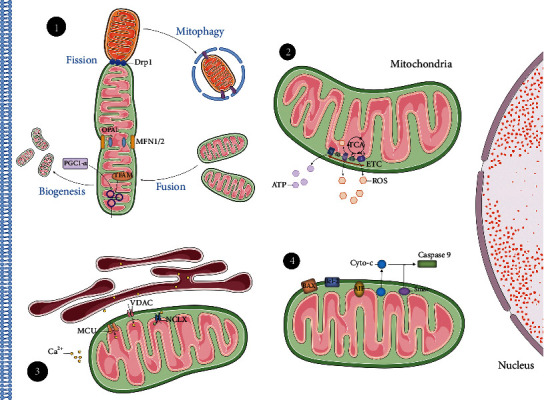
Mitochondrial dysfunction mechanism of spiral ganglion neurons in auditory neuropathy. Although mechanisms of mitochondrial dysfunction have not been illustrated distinctly, damages in following targets have been mentioned: (1) mitochondrial homeostasis including biogenesis, dynamics, and mitophagy; (2) redox homeostasis and energetic metabolism; (3) mitochondrial calcium homeostasis; and (4) proapoptotic signal in mitochondria. Drp1: dynamin-related protein 1; MFN1/2: mitofusin 1/2; OPA1: optic atrophy 1; PGC1-*α*: peroxisome proliferator-activated receptor *γ* coactivator-1 *α*; TFAM: mitochondrial transcription factor A; TCA: tricarboxylic acid; ETC: electron transport chain; MCU: mitochondrial calcium uniporter; VDAC: voltage-dependent anion channel; NCLX: Na+/Ca2+/Li+ exchanger; Bcl-2: B cell lymphoma-2; BAX: Bcl-2 associated protein X; AIF: apoptosis inducing factor.

Most of SGNs are bipolar cells located in Rosenthal's canal around the modiolus, serving as the primary afferent nerves with innervation of the sensory HCs and cochlear nucleus [38, 39]. About 95% of SGNs embedded in myelin formed by satellite glial cells are connected to IHCs, named type I SGNs [40]. The rest of the neurons are type II SGNs and act as postsynaptic sites of OHCs. When action potentials of HCs were initiated by acoustic signal, glutamine, the neurotransmitters were released at ribbon synapses, which was highly specific with precise and speedy information transmission, inducing action potential of SGNs through AMPA receptors [41, 42]. Consequently, SGNs gathered sound signals from dendrites and communicated to an auditory nucleus through axon. The average length of fiber between SGN and HCs in human was nearly 32 mm [43], which required high energetic consumption and protein synthesis to complete long distance transportation [44]. Imperative requirement of energy support by mitochondria in SGNs indicated the contribution of mitochondrial dysfunction may induce auditory neuropathy (Figure 1).

### 3.2. Mitochondrial Homeostasis in Auditory Neuropathy

Deregulation of mitochondrial homeostatic mechanism might probably contribute to auditory neuropathy, with dysfunctional mitochondrion biogenesis or impaired dynamics. PGC1-*α*, a key regulator of mitochondrial biogenesis, was also found increased in HCs and auditory cortex, which might improve the sensitivity of age-related hearing loss [45–47]. Additionally, it was found that mutation of tRNA 5-methylaminomethyl-2-thiouridylate methyltransferase (TRMU), the tRNA-modified protein, was related to incidence of SNHL [48, 49]. Dysfunction on mitochondrial protein synthesis plays a fundamental role in SNHL development, when tryptophanyl-tRNA synthetase 2 (Wars2) and mitochondrial ribosomal protein S2 (MRPS2), which are critical to the process, were proved to lead to severe SNHL and SGN loss during mutation [50, 51]. Mitochondrial protein transport dysfunction also drives the development of SNHL, such as GFER, mitochondrial disulfide relay system protein [52], and DDP [53]. Performing as the critical protein of mitochondrial fission, OPA1 R455H missense mutations were also discovered linking to auditory neuropathy. The absence of ABR, serious speech perception impairment with preserved activity of OHCs, points to the damage of IHCs, ribbon synapse, or auditory nerves [54]. PINK1 is widely expressed in mouse cochlea and able to protect SGNs from cisplatin-induced ototoxicity [55]. Conversely, mitophagy deficiency due to Drp-1 inhibition might give rise to age-related hearing loss with impaired mitochondrial membrane potential HC damage [56].

### 3.3. Redox Homeostasis and Energetic Metabolism in Auditory Neuropathy

Due to abundant antioxidant enzyme and low transfer potential energy, mitochondria with integrated structure and function can defend against the formation of ROS [57]. ROS homeostasis was associated with neurodegeneration and auditory neuropathy [58]. Three-week-old mice infected with murine congenital cytomegalovirus (MCMV) in neonatal were found to be suffering from hearing loss, and MCMV-infected cultured SGNs in vitro displayed elevated ROS levels and activated NLRP3 inflammasome, which can be suppressed by ROS inhibitor NAC [59]. Additionally, ROS is related to cochlear neuropathy in presbycusis. Evaluated mtDNA oxidative damage and mitochondrial ultrastructural damage in SGNs and auditory cortex were described in aging C57/B6j mice [60]. To mimic human's presbycusis, a senescence-accelerated mouse prone 8 (SAMP8) mouse model was chosen to study the mechanism of ARHL. SGNs of SAMP8 mice own disorganized mitochondria with missing cristae at 12 months, and MDA (a lipid peroxidation) increased and antioxidant enzyme decreased in 1 month, compared to wild-type mice [61]. Disrupted CMP-Neu5Ac hydroxylase (Cmah) is also involved in ARHL. Cmah-null mice showed significant downregulation of ROS gene degradation such as Gpxs and Sod; meanwhile, SGNs lost dramatically. KEGG pathway analysis demonstrated downregulation of mitochondrial molecular transport regulator gene, including Crumbs homolog 1 (Crb1), mitochondrial fission process 1 (Mtfp1), Ras homolog family member T2 (RhoT2), soluble oxidase component (Soc2), and ATP synthase F1 (Atp5f1), indicating mitochondrial dysfunction [62]. The mutation of the protein that can affect ROS production and degradation such as superoxide dismutase (SOD) [63], glutathione S-transferases (GST) [64], mitochondrial uncoupling proteins (UCPs) [65] were found be associated with ARHL.

Now, we have consensus that excessive ROS production aroused cochlear injury in NIHL [66, 67]. Noise exposure induced ROS damage, and raised mitochondrial calcium leads to endoplasmic reticulum (ER) and extracellular fluid, which damage abnormal mitochondrial membrane potential [68–70]. The stria vascularis also contributed to neuropathy: lipid peroxide formation and swollen blood vessels in stria vascularis reduced cochlear blood flow [71, 72], resulting in cochlea ischemia reperfusion and secondary injury by ROS. Noise exposure also caused glutamate excitotoxic neural swelling [67, 73]. A previous study of excessive ROS production after noise exposure focus on the HCs rather than SGN. Although it was still unknown whether ROS was associated with synapse and SGNs damage in NIHL, SGN was susceptive to hypoxia demonstrated by patients who experienced perinatal and postnatal hypoxia [74].

TCA cycle is a key process for energy-intensive auditory nerves. Isocitrate dehydrogenase 2 (IDH2) is one of the isozymes of IDH and can convert NADP+ to NADPH, involved in TCA cycle. IDH2 dysfunction accelerated apoptosis and caused cardiac impairment due to oxidative stress [75, 76]. Severe oxidative damage and more fragmented nuclear DNA in SGNs were seen in Idh2^−/−^ mice at 24 months compared to WT, indicating IDH2 deficiency promotes age-related hearing loss [77]. Calorie restriction protected HC and SGN degeneration by the promotion of mitochondrial antioxidant defense with sirtuin 3 (Sirt3), which boosted longevity and hearing maintenance [78]. Besides, Sirt3 and Sirt1 help inhibit p53 and restrain apoptosis [79].

### 3.4. Calcium Homeostasis in Auditory Neuropathy

Calcium ions (Ca^2+^) are secondary messengers in many crucial cellular activities, for instance, cell death and organ development. To maintain proper Ca^2+^ signaling, a mitochondrion is a vital mediator of calcium in ER, the major intracellular Ca^2+^ pool. Mitochondrion-associated ER membranes (MAMs), referring to ER-mitochondrion connection, possess calcium transport proteins and channels [80]. MAMs permit fast calcium flux between ER and mitochondrial matrix, which is essential for neural excitation. After being released by ER, calcium ions traverse voltage-dependent anion-selective channel (VDAC) and mitochondrial calcium uniporter (MCU) located in the bilayer of mitochondria and can be extruded to the cytoplasm by sodium calcium exchanger (NCLX) [81]. MCU regulates the activity of enzymes in the TCA cycle [82] and sensitivity of synapses in cochlea to noise exposure. MCU was found to be increased in HCs after noise. Treatment with MCU siRNA or specific MCU inhibitor Ru360 alleviated HCs and ribbon synapse degeneration after noise into CBA/J mice. MCU inhibition reduced ABR wave I amplitude damage, suggesting that MCU was correlated to cochlear synaptopathy [83]. Moreover, superfluous calcium uptake results in swollen mitochondria and abnormal mitochondrial membrane potential, inducing mitochondrial apoptotic factors released to the cytoplasm [84].

### 3.5. Apoptosis in Neuropathy

Mitochondria are of great importance to induce apoptosis under intrinsic and extrinsic stimulations by means of proapoptotic signal like activation of BH3-only protein or calcium influx and releasing apoptotic protein including cyto-c, caspases, AIF, and Smac [85, 86]. Abnormal mitochondrial might cause apoptosis in cochlear nerves. Apoptosis-inducing factor (AIF), a flavoprotein and redox enzyme located in mitochondrial intermembrane which can condense chromatin and fracture DNA, was found to be activated by glutamate, which resulted in SGN apoptosis. Calpain was proved to promote mature AIF [87]. Pyridoxine damaged nerve fiber by inducing overload of mitochondrial calcium and activation of apoptosis signal from Bcl-2 family ROS generation and mitochondrial potential transition (MPT) were also aroused after pyridoxine treatment [88]. Although overexpression of bcl-2 might inhibit SGN apoptosis, growth of SGN neurite was suppressed in vitro [89].

## 4. Therapy in Auditory Neuropathy

**Figure 2:**
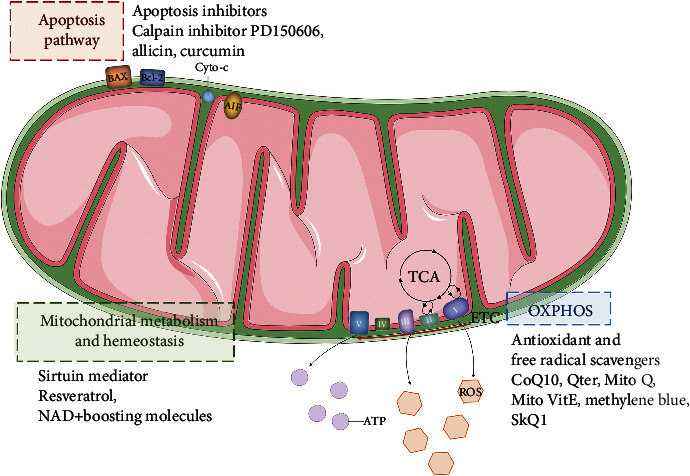
Pharmacological targets of mitochondria in auditory neuropathy. Pharmacological therapeutics of mitochondrial dysfunction to rescue auditory neuropathies are still limited. Proven therapeutic strategy targets are comprised of apoptosis inhibition, sirtuin mediators maintaining mitochondrial homeostasis and capability of metabolism, and antioxidants and free radical scavengers that are helpful to alleviate oxidative stress.

With the intensive study of mechanism between mitochondrial dysfunction and auditory neuropathy, novel perspectives of mitochondrion-targeted therapies were explored. There were several therapies targeting mitochondrial, which will rescue auditory neuropathy fundamentally (Figure 2).

### 4.1. Antioxidants

Antioxidants were elucidated to protect SNHL by eliminating excessive ROS products, including an intrinsic system such as SODs and GSH and extrinsic system such as inhibitors of calcium, HSP, or salicylate [90].

CoQ10, a common redox in mitochondria and cofactor of respiratory chain, has the capacity of permitting electron and proton transport through ETC and debriding ROS as the antioxidant [91]. Supplementation of water-soluble coenzyme Q10 analog (Qter) alleviated damage of SGNs after noise exposure [92] as well as prevented presbycusis in murine [93].

Methylene blue (MB), distinguished as histological dye, was first applied to clinical practice for the treatment of malaria. Besides, MB could also prevent mitochondria from overproduction of ROS by rerouting electron from NADH to cyto-c and was proved beneficial to neurodegeneration covering NIHL, AD, and PD [94]. Pretreatment with MB diminished ROS and evaluated neurotrophin-3 (NT-3) level, protecting nerve terminals between HCs and SGNs from NIHL [95].

The limitation of the antioxidants was distinct that they could not sweep up ROS in mitochondria precisely and effectively. Recently, studies have shown mitochondrion-targeted antioxidant MitoQ concentrated in solving conventional antioxidant could not aggregate precedingly [96]. MitoQ comprises CoQ10 and lipophilic triphenyl phosphonium (TPP), endowing CoQ10 with the ability to go through a phospholipid bilayer and gather inside mitochondria rapidly, which could stabilized mitochondrial function by enhancing mitochondrial fusion via activation of PGC1-*α* and upregulation of Mfn2 in the PD model [97]. Besides, other mitochondrion-targeted antioxidants like Mito VitE, and SkQ1 were developed, while the therapeutic effect to auditory neuropathy required verification [98].

### 4.2. Sirtuin Mediators

Sirtuins are from NAD+-dependent deacylase family which is of great importance to aging and nervous system. SIRT1 participates in the regulation of cellular ROS, synaptic plasticity, and extending lifespan in collaboration with SIRT3, the modulator of mitochondrial metabolism [99]. Sirtuin mediators like resveratrol and NAD+ supplement are also popular in antiaging [100], which were also found efficient in NIHL [101, 102]. Resveratrol, an activator of SIRT1, is a natural antioxidant relevant to mitochondrial biogenesis and modification of mitochondrial function. Mitigatory SGN degeneration and enhancive expression of PINK and Parkin were observed in the mice with long-term replenishment, revealing intensive mitophagy but improved mitochondrial function [100]. Additionally, resveratrol was able to eliminate toxicity protein SGNs from injury caused by noise exposure [101].

NAD, as key coenzyme in several cellular events, took part in the crucial process in mitochondrial metabolism and was associated with axonal degenerations and neurodegeneration. Supplementation of NAD could protect damage neurons and delay neurodegeneration [103]. In hearing loss induced by Mn, NAD was suggested to prevent auditory nerve fibers and SGNs from axonal degeneration and cell apoptosis [102].

### 4.3. Apoptosis Inhibitors

Due to apoptosis induced by mitochondrial dysfunction, inhibitors of apoptosis targeting mitochondria were developed and found efficient to SNHL. A calpain inhibitor PD150606 could suppress calpain by mediating AIF induced by glutamine and caspase-12 activation, restraining apoptosis processing and SGNs in vitro [87]. Meanwhile, allicin [104] and curcumin [105] were found to protect SGNs from ototoxic drugs, when paeoniflorin and neurotrophin might exert as protective effect through the PINK1/BAD pathway [89, 106].

Others such as gene therapy [107] and stem cell therapy [108] still have been studied. But auditory neuropathy treatment is still limited, requiring more exploration.

## 5. Conclusion

Mitochondrial dysfunction was demonstrated to involve in both hereditary and acquired hearing loss, and the mechanism of ROS damage and mutation of mtDNA in HC were studied intensively. Mitochondrion function as the energy manufacturer and regulator of apoptosis and calcium homeostasis, which is able to induce SGN damage. The function of mitochondria and the association to neurodegeneration have been excavated, extending perspective on the relationship between mitochondrial dysfunction and auditory neuropathy. Here, we summarized the association between auditory neuropathy and mitochondrial dysfunction of SGNs, as well as therapeutics targeting mitochondria in AN. Treatments of optic neuropathy including drugs, gene, and stem cell therapies [109] inspired us to explore effective therapeutics for AN.
